# Thank you to our retiring editors Anke Becker and Conrad Mullineaux

**DOI:** 10.1128/jb.00309-25

**Published:** 2025-08-11

**Authors:** George A. O’Toole

**Affiliations:** 1 Department of Microbiology and Immunology, Geisel School of Medicine at Dartmouth, Hanover, New Hampshire, USA; University of Notre Dame, Notre Dame, Indiana, USA

**Keywords:** microbial, community, model, mechanism

## EDITORIAL

When you hear “scientific journal,” what do you think about? Some amorphous organization? A data farm where all the digital information is stored? Or for those a bit older, the rows and rows of paper journals in your advisor’s office? When I think about this enterprise, I think about people—all the people involved in taking your submitted manuscript and turning it into a published paper. We celebrate our editorial board by putting them on the journal masthead and *ad hoc* reviewers with an annual editorial. Today, I would like to celebrate the retirement of two of our esteemed editors: Anke Becker and Conrad Mullineaux.

Anke works at the Center for Synthetic Microbiology, Philipps-Universität Marburg, Fachbereich Biologie in Marburg, Germany. To give a sense of the breadth of topics she covers in her role, here is a list of the key words used to assign her papers: bacteria–plant interactions, symbiosis, polysaccharides, gene expression, second messengers, quorum sensing, megaplasmids, chromids, alphaproteobacterial, Rhizobium, genomics, and transcriptomics. Anke served on the editorial board of the *Journal of Bacteriology* (JB) from 2010 to 2015 and was appointed as an editor in 2015. She is retiring after 10 years of service.

Conrad is in the School of Biological and Behavioral Sciences at Queen Mary University of London in England. He equally covers a lot of ground at JB with these key words: cyanobacteria, *Escherichia coli*, membranes, membrane biophysics, photosynthetic accessory pigments, phototrophs, electron transport, respiration, respiratory oxidases, bioenergetics, protein localization, biophysical approaches, microscopy, phototaxis, type IV pili cell–cell interaction, and biofilms. Again, you can see we look for editors with broad expertise. Conrad served as an editorial board member from 2008 to 2015 and as an editor from 2015 to 2025.

Anke and Conrad together handled almost 900 of your manuscripts during their time as editors and typically delivered a decision to an author within 3 weeks or less after submission. This is a massive effort for which I am very grateful! While Anke and Conrad will be stepping away from their role as editors, I am sure we will still be tapping their expertise as reviewers—one’s work is never done! So, when I equate the words “scientific journal” to people, it is people like Anke and Conrad that come to mind.

Thank you for all your service to JB!

